# Homopiperazine-1,4-diium bis­[hexa­aqua­cobalt(II)] tris­ulfate

**DOI:** 10.1107/S1600536811027012

**Published:** 2011-07-13

**Authors:** Thameur Sahbani, Wajda Smirani Sta, Salem S. Al-Deyab, Mohamed Rzaigui

**Affiliations:** a Laboratoire de Chimie des Matériaux, Faculté des Sciences de Bizerte, 7021 Zarzouna Bizerte, Tunisia; b Petrochemical Research Chair, College of Science, King Saud University, Riyadh, Saudi Arabia

## Abstract

In the title compound, (C_5_H_14_N_2_)[Co(H_2_O)_6_]_2_(SO_4_)_3_, the cationic framework is built up of mixed organic–inorganic fragments, namely [Co(H_2_O)_6_]^2+^ and [C_5_H_14_N_2_]^2+^. The [Co(H_2_O)_6_]^2+^ cations form unconnected octa­hedra. Sulfate anions inter­calated between cationic species connect them *via* N—H⋯O and O—H⋯O hydrogen bonds and electrostatic inter­actions.

## Related literature

For sulfate chemistry with amines, see: Bataille & Louer (2002 ▶, 2004 ▶); Xing *et al.* (2003 ▶); Morimoto & Lingafelter (1970 ▶). For related structures, see: Hemissi *et al.* (2010 ▶); Rekik *et al.* (2009 ▶); Wilkinson & Harrison (2006 ▶); Pan *et al.* (2003 ▶).
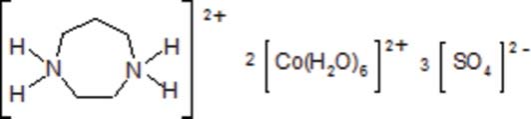


## Experimental

### 

#### Crystal data

(C_5_H_14_N_2_)[Co(H_2_O)_6_]_2_(SO_4_)_3_
*M* *_r_* = 724.41
Monoclinic, 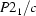

*a* = 14.109 (2) Å
*b* = 11.730 (3) Å
*c* = 16.696 (5) Å
β = 106.65 (2)°
*V* = 2647.2 (11) Å^3^
*Z* = 4
Ag *K*α radiation
λ = 0.56085 Å
μ = 0.83 mm^−1^
*T* = 293 K
0.30 × 0.25 × 0.20 mm

#### Data collection

Enraf–Nonius TurboCAD-4 diffractometer
16044 measured reflections
12932 independent reflections
6008 reflections with *I* > 2σ(*I*)
*R*_int_ = 0.044
2 standard reflections every 120 min  intensity decay: 5%

#### Refinement

*R*[*F* ^2^ > 2σ(*F* ^2^)] = 0.064
*wR*(*F* ^2^) = 0.163
*S* = 0.98
12932 reflections
325 parameters
H-atom parameters constrained
Δρ_max_ = 0.99 e Å^−3^
Δρ_min_ = −1.08 e Å^−3^

### 

Data collection: *CAD-4 EXPRESS* (Enraf–Nonius, 1994 ▶); cell refinement: *CAD-4 EXPRESS*; data reduction: *XCAD4* (Harms & Wocadlo, 1995 ▶); program(s) used to solve structure: *SHELXS97* (Sheldrick, 2008 ▶); program(s) used to refine structure: *SHELXL97* (Sheldrick, 2008 ▶); molecular graphics: *ORTEPIII* (Burnett & Johnson, 1996 ▶) and *ORTEP-3 for Windows* (Farrugia, 1997 ▶); software used to prepare material for publication: *WinGX* (Farrugia, 1999 ▶).

## Supplementary Material

Crystal structure: contains datablock(s) I, global. DOI: 10.1107/S1600536811027012/dn2704sup1.cif

Structure factors: contains datablock(s) I. DOI: 10.1107/S1600536811027012/dn2704Isup2.hkl

Additional supplementary materials:  crystallographic information; 3D view; checkCIF report

## Figures and Tables

**Table 1:** Hydrogen-bond geometry (Å, °)

*D*—H⋯*A*	*D*—H	H⋯*A*	*D*⋯*A*	*D*—H⋯*A*
N1—H1*C*⋯O24	0.90	1.87	2.758 (4)	171
N1—H1*D*⋯O18^i^	0.90	1.84	2.723 (4)	167
N2—H2*C*⋯O15	0.90	2.00	2.820 (4)	151
N2—H2*D*⋯O21^ii^	0.90	1.99	2.850 (4)	161
O1—H11⋯O23^iii^	0.85	1.91	2.738 (3)	165
O1—H12⋯O20	0.85	1.93	2.774 (3)	178
O2—H21⋯O19^iv^	0.84	1.84	2.682 (4)	176
O2—H22⋯O15	0.85	2.00	2.843 (4)	169
O3—H31⋯O24^v^	0.85	1.89	2.722 (3)	167
O3—H32⋯O16	0.85	2.16	2.982 (4)	163
O3—H32⋯O15	0.85	2.53	3.152 (4)	130
O4—H41⋯O20^vi^	0.85	1.99	2.840 (3)	175
O4—H42⋯O17	0.85	1.90	2.733 (3)	166
O5—H51⋯O17^iv^	0.86	2.00	2.855 (3)	175
O5—H52⋯O23^v^	0.86	1.88	2.726 (3)	169
O6—H61⋯O19^vi^	0.85	1.82	2.665 (4)	178
O6—H62⋯O22^iii^	0.85	1.92	2.749 (3)	164
O7—H71⋯O15	0.84	2.14	2.908 (4)	152
O7—H72⋯O17	0.84	2.00	2.829 (3)	169
O8—H81⋯O16	0.85	1.88	2.722 (3)	174
O8—H82⋯O13^vii^	0.85	1.88	2.725 (3)	171
O9—H91⋯O18	0.85	1.84	2.681 (3)	173
O9—H92⋯O14^viii^	0.85	1.91	2.742 (4)	165
O10—H101⋯O22^vii^	0.85	1.94	2.788 (3)	174
O10—H102⋯O14^vii^	0.84	1.91	2.742 (3)	170
O11—H111⋯O21^ii^	0.85	1.92	2.759 (3)	168
O11—H112⋯O16^viii^	0.85	1.88	2.729 (3)	174
O12—H121⋯O21^vii^	0.85	1.96	2.806 (3)	176
O12—H122⋯O13	0.85	1.91	2.728 (3)	162

Symmetry codes: (i) 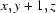; (ii) 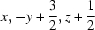; (iii) 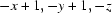; (iv) 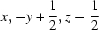; (v) 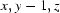; (vi) 
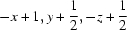
; (vii) 
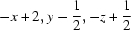
; (viii) 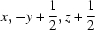.

**Fig. 1.**
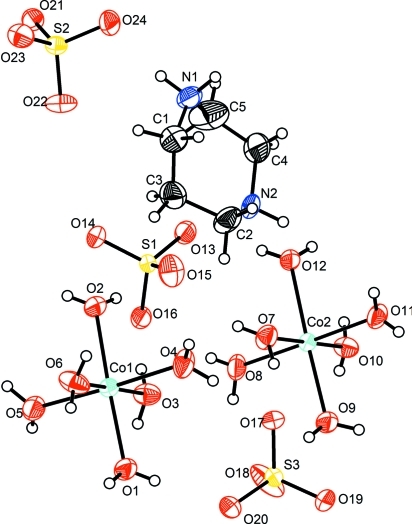
The assymetric unit of the title compound, with the atom numbering scheme. Displacement ellipsoids are drawn at the 30% probability level. H atoms are represented as small sphere of arbitrary radii. Hydrogen bonds are shown as dashed lines.

**Fig. 2.**
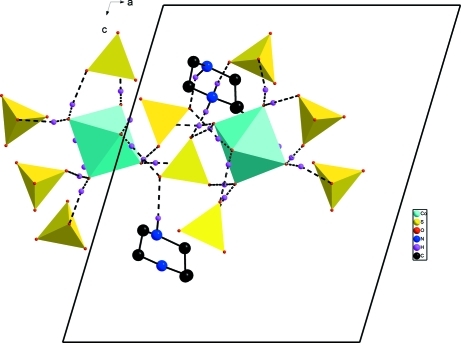
Connection of the different entities via H-bonds into supramolecular network. Hydrogen bonds are shown as dashed lines.

## supplementary crystallographic information

### Comment

The sulfate chemistry has regained interest for a few years, mainly with the idea of using amines as templates in hydrothermal syntheses, and also because of the tetrahedral shape of using amines as templates in hydrothermal syntheses SO_4_^2-^ that promises to extend the zeotype materials to sulfate compounds (Bataille & Louer, 2002). Many structures have been obtained, with one-dimensional (Bataille & Louer, 2004), two-dimensional (Xing *et al.*, 2003)and three-dimensional (Morimoto & Lingafelter, 1970)architectures, consisting of inorganic frameworks built up from strong metal–oxygen bonds. By cons, supramolecular networks of hexaaquametal sulfates including amine groups have been much less investigated. While the crystal engineering of supramolecular compounds is known to favour electric, magnetic and optical properties, supramolecular compounds containing sulfate are still relatively few. We report herein a novel cobalt sulfate template by homopiperazine, (C_5_H_14_N_2_) (Co(H_2_O)_6_)_2_ (SO_4_)_3_ (I).

The crystal structure of (C_5_H_14_N_2_)(Co(H_2_O)_6_)_2_(SO_4_)_3_ has an asymmetric unit consisting of two cobalt cations octahedrally coordinate by six water molecules, [Co(H_2_O)_6_]^2+^, three isolated sulfate anions, SO_4_^2-^, and one diprotonated homopiperazine cation, C_5_H_14_N_2_^2+^ (Fig.1). These components are linked together by hydrogen bonds to form a three-dimensional supramolecular network (Fig.2).

In this compound, the cobalt atoms occupy general positions and are at the centre of slightly distored octahedron formed by six water molecules. Within these octahedra, the Co—Ow distances range from 2.057 (3) to 2.106 (3) and from 2.061 (2) to 2.135 (3) Å, for Co^2+^(1) and Co^2+^(2), respectively. The values of Ow—Co—Ow angles are between 84.67 (11) and 177.76 (11)° in the Co^2+^(1) octahedron and between 84.93 (11) and 178.98 (12)° in the Co^2+^(2) octahedron. These geometrical characteristics agree with those described in the literature for cobalt octahedron formed by six water molecules too (Pan *et al.*, 2003; Rekik *et al.*, 2009). The Co(H_2_O)_6_ octahedra are separated from each other with the shortest cobalt–cobalt distance being 6.308 Å. In this structure each Co^2+^ octahedron is surrounded by six sulfate anions, connected *via* hydrogen bonds in a bidentate fashion. Only one homopiperazinium cation exists in the asymmetric unit and adopts a chair conformation as evidenced by the mean deviation (± 0.027) from the least square plane. A similar conformation for the same organic molecule was observed in (C_5_H_14_N_2_)(H_2_AsO_4_)_2_ (Wilkinson & Harrison, 2006).

There are three independent SO_4_^2-^ anions in this structure. The geometrical characteristics of these anions are comparable and are not very distinct from these observed in other compounds containing the same group (Hemissi *et al.*, 2010). These sulfate anions compensate the positive charges of the bis-hexaaquacobalt (II) and ensure the cohesion of the packing. . Indeed, all oxygen atoms of the SO_4_ groups participate as acceptor in hydrogen bonds accepting hydrogen atoms of the organic moiety and the complex Co(H_2_O)_6_^2+^. This kind of bonds participates with other interactions (namly electrostatic and Van Der Waals) to form a stable three-dimensional network.

### Experimental

Single crystals of the title compound, (C_5_H_14_N_2_)(Co(H_2_O)_6_)_2_(SO_4_)_3_, were prepared by adding ethanolic solution (5 ml) of homopiperazine (5 mmol) dropwise to an aqueous solution of cobalt sulfate Co(SO_4_).7H_2_O (10 mmol, 10 ml). The obtained mixture was added to an aqueous solution of sulfuric acid (15 mmol, 20 ml). The clear solution were slowly stirred for 20 min and allowed to stand at room temperature (293 K) untill single pink crystals were formed.

### Refinement

The aqua H atoms were located in a difference map. H atoms bonded to C and N atoms were positioned geometrically were positioned geometrically and treated as riding on their parent atoms, [N–H = 0.89, C–H =0.96 Å (CH_3_ ) with with *U*_iso_(H) = 1.5Ueq and C–H =0.96 Å (Ar–H), with *U*_iso_(H) = 1.5Ueq]

### Crystal data

**Table:**

(C_5_H_14_N_2_)[Co(H_2_O)_6_]_2_(SO_4_)_3_	*F*(000) = 1504
*M**_r_* = 724.41	*D*_x_ = 1.818 Mg m^−^^3^
Monoclinic, *P*2_1_/*c*	Ag *K*α radiation, λ = 0.56085 Å
Hall symbol: -P 2ybc	Cell parameters from 25 reflections
*a* = 14.109 (2) Å	θ = 9–11°
*b* = 11.730 (3) Å	µ = 0.83 mm^−^^1^
*c* = 16.696 (5) Å	*T* = 293 K
β = 106.65 (2)°	Block, pink
*V* = 2647.2 (11) Å^3^	0.30 × 0.25 × 0.20 mm
*Z* = 4	

### Data collection

**Table:**

Enraf–Nonius TurboCAD-4 diffractometer	*R*_int_ = 0.044
Radiation source: fine-focus sealed tube	θ_max_ = 28.0°, θ_min_ = 2.0°
graphite	*h* = −23→22
Non–profiled ω scans	*k* = −2→19
16044 measured reflections	*l* = −1→27
12932 independent reflections	2 standard reflections every 120 min
6008 reflections with *I* > 2σ(*I*)	intensity decay: 5%

### Refinement

**Table:**

Refinement on *F*^2^	Primary atom site location: structure-invariant direct methods
Least-squares matrix: full	Secondary atom site location: difference Fourier map
*R*[*F*^2^ > 2σ(*F*^2^)] = 0.064	Hydrogen site location: inferred from neighbouring sites
*wR*(*F*^2^) = 0.163	H-atom parameters constrained
*S* = 0.98	*w* = 1/[σ^2^(*F*_o_^2^) + (0.0669*P*)^2^] where *P* = (*F*_o_^2^ + 2*F*_c_^2^)/3
12932 reflections	(Δ/σ)_max_ = 0.001
325 parameters	Δρ_max_ = 0.99 e Å^−^^3^
0 restraints	Δρ_min_ = −1.08 e Å^−^^3^

### Special details

**Table:**

Geometry. All esds (except the esd in the dihedral angle between two l.s. planes) are estimated using the full covariance matrix. The cell esds are taken into account individually in the estimation of esds in distances, angles and torsion angles; correlations between esds in cell parameters are only used when they are defined by crystal symmetry. An approximate (isotropic) treatment of cell esds is used for estimating esds involving l.s. planes.
Refinement. Refinement of *F*^2^ against ALL reflections. The weighted *R*-factor *wR* and goodness of fit *S* are based on *F*^2^, conventional *R*-factors *R* are based on *F*, with *F* set to zero for negative *F*^2^. The threshold expression of *F*^2^ > σ(*F*^2^) is used only for calculating *R*-factors(gt) *etc*. and is not relevant to the choice of reflections for refinement. *R*-factors based on *F*^2^ are statistically about twice as large as those based on *F*, and *R*- factors based on ALL data will be even larger.

### Fractional atomic coordinates and isotropic or equivalent isotropic displacement parameters (Å^2^)

**Table:**

	*x*	*y*	*z*	*U*_iso_*/*U*_eq_	
C1	0.6475 (3)	0.7803 (3)	0.1987 (3)	0.0438 (9)	
H1A	0.6081	0.8003	0.2357	0.053*	
H1B	0.6124	0.8077	0.1434	0.053*	
C2	0.6837 (3)	0.5952 (4)	0.2792 (3)	0.0493 (10)	
H2A	0.6528	0.6333	0.3168	0.059*	
H2B	0.6590	0.5176	0.2719	0.059*	
C3	0.6529 (3)	0.6551 (4)	0.1953 (3)	0.0491 (10)	
H3A	0.6994	0.6348	0.1647	0.059*	
H3B	0.5885	0.6264	0.1639	0.059*	
C4	0.8416 (3)	0.7049 (4)	0.3319 (3)	0.0664 (14)	
H4A	0.8154	0.7480	0.3704	0.080*	
H4B	0.9113	0.6923	0.3591	0.080*	
C5	0.8332 (3)	0.7752 (4)	0.2587 (4)	0.0730 (16)	
H5A	0.8397	0.7259	0.2139	0.088*	
H5B	0.8889	0.8273	0.2715	0.088*	
Co1	0.56846 (3)	0.25194 (4)	0.08643 (2)	0.02460 (9)	
Co2	0.94025 (3)	0.24877 (4)	0.40481 (2)	0.02251 (9)	
N1	0.7432 (2)	0.8420 (3)	0.2272 (2)	0.0404 (7)	
H1C	0.7498	0.8842	0.1840	0.048*	
H1D	0.7393	0.8909	0.2677	0.048*	
N2	0.7916 (2)	0.5921 (3)	0.31858 (16)	0.0354 (6)	
H2C	0.8193	0.5495	0.2864	0.042*	
H2D	0.8034	0.5570	0.3684	0.042*	
O1	0.52421 (17)	0.0882 (2)	0.10741 (15)	0.0378 (6)	
H11	0.4658	0.0739	0.0776	0.057*	
H12	0.5326	0.0633	0.1566	0.057*	
O2	0.62291 (18)	0.4116 (2)	0.06991 (15)	0.0371 (6)	
H21	0.6222	0.4355	0.0220	0.056*	
H22	0.6811	0.4136	0.1031	0.056*	
O3	0.71109 (16)	0.1944 (2)	0.14899 (15)	0.0364 (6)	
H31	0.7212	0.1270	0.1347	0.055*	
H32	0.7584	0.2360	0.1434	0.055*	
O4	0.5609 (2)	0.3149 (2)	0.20264 (15)	0.0451 (7)	
H41	0.5283	0.3739	0.2082	0.068*	
H42	0.5721	0.2755	0.2473	0.068*	
O5	0.57856 (19)	0.1958 (2)	−0.03016 (15)	0.0410 (6)	
H51	0.5925	0.2372	−0.0677	0.061*	
H52	0.6125	0.1348	−0.0284	0.061*	
O6	0.42671 (17)	0.3068 (2)	0.02907 (17)	0.0432 (6)	
H61	0.4143	0.3709	0.0477	0.065*	
H62	0.3749	0.2664	0.0213	0.065*	
O7	0.79520 (16)	0.3042 (2)	0.33784 (14)	0.0354 (5)	
H71	0.7881	0.3225	0.2877	0.053*	
H72	0.7495	0.2619	0.3443	0.053*	
O8	0.9567 (2)	0.1818 (2)	0.29491 (14)	0.0437 (7)	
H81	0.9392	0.2135	0.2474	0.066*	
H82	0.9692	0.1118	0.2894	0.066*	
O9	0.89302 (17)	0.0900 (2)	0.43047 (15)	0.0374 (6)	
H91	0.8367	0.0716	0.3985	0.056*	
H92	0.8957	0.0760	0.4810	0.056*	
O10	1.08348 (15)	0.19433 (19)	0.46827 (14)	0.0316 (5)	
H101	1.1329	0.2347	0.4673	0.047*	
H102	1.0911	0.1261	0.4559	0.047*	
O11	0.92294 (18)	0.3187 (2)	0.51302 (14)	0.0373 (6)	
H111	0.8898	0.3802	0.5077	0.056*	
H112	0.9133	0.2798	0.5528	0.056*	
O12	0.98677 (16)	0.41338 (19)	0.38252 (13)	0.0310 (5)	
H121	1.0375	0.4311	0.4222	0.047*	
H122	0.9990	0.4271	0.3365	0.047*	
O13	0.98207 (19)	0.4652 (2)	0.22216 (15)	0.0437 (7)	
O14	0.8746 (2)	0.4821 (2)	0.08156 (14)	0.0432 (6)	
O15	0.80762 (19)	0.4370 (3)	0.19412 (16)	0.0479 (7)	
O16	0.9033 (2)	0.2994 (2)	0.14824 (14)	0.0424 (6)	
O17	0.6251 (2)	0.17788 (19)	0.33990 (15)	0.0388 (6)	
O18	0.72061 (19)	0.0123 (3)	0.33110 (19)	0.0585 (9)	
O19	0.6113 (2)	0.0105 (2)	0.41637 (15)	0.0530 (8)	
O20	0.54614 (17)	0.0078 (2)	0.26795 (14)	0.0355 (5)	
O21	0.83964 (16)	0.9666 (2)	−0.00881 (14)	0.0311 (5)	
O22	0.74559 (16)	0.8156 (2)	0.02875 (18)	0.0408 (6)	
O23	0.66393 (17)	0.9879 (2)	−0.03288 (16)	0.0391 (6)	
O24	0.77366 (19)	0.9889 (2)	0.10789 (14)	0.0390 (6)	
S1	0.89265 (6)	0.42267 (6)	0.16061 (4)	0.02494 (15)	
S2	0.75433 (5)	0.94014 (6)	0.02367 (5)	0.02542 (16)	
S3	0.62532 (6)	0.05217 (7)	0.33890 (5)	0.02640 (16)	

### Atomic displacement parameters (Å^2^)

**Table:**

	*U*^11^	*U*^22^	*U*^33^	*U*^12^	*U*^13^	*U*^23^
C1	0.0392 (19)	0.043 (2)	0.049 (2)	0.0084 (17)	0.0122 (17)	0.0027 (18)
C2	0.043 (2)	0.055 (3)	0.050 (2)	−0.0135 (19)	0.0141 (18)	0.014 (2)
C3	0.045 (2)	0.049 (2)	0.045 (2)	−0.0027 (19)	0.0006 (18)	−0.0013 (19)
C4	0.064 (3)	0.069 (3)	0.043 (2)	−0.023 (3)	−0.022 (2)	0.012 (2)
C5	0.045 (2)	0.069 (3)	0.104 (4)	−0.020 (2)	0.019 (3)	0.011 (3)
Co1	0.02776 (19)	0.02164 (19)	0.02410 (19)	0.00090 (17)	0.00694 (15)	0.00093 (17)
Co2	0.02656 (18)	0.02121 (18)	0.01983 (17)	−0.00148 (17)	0.00676 (14)	0.00051 (16)
N1	0.0549 (19)	0.0292 (15)	0.0420 (17)	0.0006 (14)	0.0219 (15)	0.0051 (13)
N2	0.0460 (16)	0.0365 (16)	0.0223 (13)	0.0025 (14)	0.0077 (12)	0.0077 (12)
O1	0.0406 (13)	0.0365 (13)	0.0318 (12)	−0.0123 (11)	0.0033 (10)	0.0062 (11)
O2	0.0455 (14)	0.0309 (13)	0.0322 (12)	−0.0101 (11)	0.0066 (11)	0.0041 (10)
O3	0.0308 (12)	0.0270 (12)	0.0489 (15)	0.0021 (10)	0.0073 (11)	0.0000 (11)
O4	0.0683 (18)	0.0411 (15)	0.0299 (13)	0.0241 (14)	0.0203 (12)	0.0061 (11)
O5	0.0640 (17)	0.0290 (13)	0.0336 (13)	0.0116 (12)	0.0199 (12)	0.0038 (11)
O6	0.0328 (12)	0.0296 (13)	0.0599 (17)	0.0037 (11)	0.0015 (12)	−0.0117 (12)
O7	0.0306 (11)	0.0423 (14)	0.0298 (12)	−0.0032 (11)	0.0030 (9)	0.0083 (11)
O8	0.0777 (19)	0.0295 (13)	0.0240 (11)	0.0162 (13)	0.0147 (12)	0.0012 (10)
O9	0.0418 (13)	0.0352 (13)	0.0313 (12)	−0.0143 (11)	0.0042 (10)	0.0031 (11)
O10	0.0279 (11)	0.0249 (11)	0.0390 (13)	−0.0002 (9)	0.0050 (10)	−0.0015 (10)
O11	0.0582 (16)	0.0319 (13)	0.0277 (12)	0.0113 (12)	0.0217 (11)	0.0052 (10)
O12	0.0341 (11)	0.0331 (12)	0.0240 (10)	−0.0109 (10)	0.0054 (9)	0.0014 (9)
O13	0.0488 (15)	0.0461 (16)	0.0283 (12)	−0.0225 (13)	−0.0017 (11)	0.0059 (11)
O14	0.0696 (18)	0.0322 (13)	0.0225 (11)	−0.0079 (13)	0.0047 (12)	0.0063 (10)
O15	0.0392 (14)	0.066 (2)	0.0419 (15)	0.0050 (14)	0.0169 (12)	−0.0046 (14)
O16	0.0789 (19)	0.0238 (12)	0.0272 (12)	0.0069 (13)	0.0197 (12)	−0.0008 (10)
O17	0.0590 (16)	0.0233 (11)	0.0326 (13)	−0.0089 (11)	0.0106 (12)	0.0022 (10)
O18	0.0319 (13)	0.071 (2)	0.071 (2)	−0.0068 (14)	0.0114 (13)	−0.0414 (17)
O19	0.102 (2)	0.0343 (14)	0.0258 (12)	−0.0163 (15)	0.0233 (14)	0.0021 (11)
O20	0.0375 (13)	0.0345 (13)	0.0289 (12)	−0.0097 (11)	0.0007 (10)	−0.0023 (10)
O21	0.0300 (11)	0.0347 (13)	0.0310 (11)	−0.0062 (10)	0.0127 (9)	−0.0021 (10)
O22	0.0342 (13)	0.0215 (11)	0.0671 (18)	−0.0031 (10)	0.0150 (12)	0.0015 (12)
O23	0.0332 (12)	0.0325 (13)	0.0439 (15)	0.0063 (11)	−0.0014 (11)	0.0009 (11)
O24	0.0532 (15)	0.0383 (14)	0.0278 (12)	0.0034 (12)	0.0150 (11)	−0.0033 (11)
S1	0.0341 (4)	0.0218 (3)	0.0176 (3)	−0.0048 (3)	0.0053 (3)	−0.0009 (3)
S2	0.0250 (3)	0.0205 (3)	0.0309 (4)	0.0007 (3)	0.0083 (3)	−0.0009 (3)
S3	0.0321 (4)	0.0255 (4)	0.0209 (3)	−0.0059 (3)	0.0064 (3)	−0.0019 (3)

### Geometric parameters (Å, °)

**Table:**

C1—C3	1.472 (5)	O2—H21	0.8449
C1—N1	1.486 (5)	O2—H22	0.8494
C1—H1A	0.9700	O3—H31	0.8491
C1—H1B	0.9700	O3—H32	0.8530
C2—N2	1.476 (5)	O4—H41	0.8504
C2—C3	1.515 (5)	O4—H42	0.8533
C2—H2A	0.9700	O5—H51	0.8587
C2—H2B	0.9700	O5—H52	0.8573
C3—H3A	0.9700	O6—H61	0.8500
C3—H3B	0.9700	O6—H62	0.8494
C4—C5	1.450 (6)	O7—H71	0.8426
C4—N2	1.486 (5)	O7—H72	0.8447
C4—H4A	0.9700	O8—H81	0.8465
C4—H4B	0.9700	O8—H82	0.8513
C5—N1	1.457 (5)	O9—H91	0.8482
C5—H5A	0.9700	O9—H92	0.8494
C5—H5B	0.9700	O10—H101	0.8470
Co1—O6	2.058 (2)	O10—H102	0.8411
Co1—O2	2.072 (2)	O11—H111	0.8505
Co1—O1	2.080 (2)	O11—H112	0.8482
Co1—O3	2.096 (2)	O12—H121	0.8494
Co1—O5	2.098 (2)	O12—H122	0.8503
Co1—O4	2.107 (2)	O13—S1	1.467 (2)
Co2—O11	2.061 (2)	O14—S1	1.449 (2)
Co2—O9	2.064 (2)	O15—S1	1.472 (3)
Co2—O8	2.069 (2)	O16—S1	1.474 (2)
Co2—O10	2.094 (2)	O17—S3	1.475 (2)
Co2—O12	2.107 (2)	O18—S3	1.464 (3)
Co2—O7	2.133 (2)	O19—S3	1.448 (3)
N1—H1C	0.9000	O20—S3	1.471 (2)
N1—H1D	0.9000	O21—S2	1.488 (2)
N2—H2C	0.9000	O22—S2	1.471 (2)
N2—H2D	0.9000	O23—S2	1.463 (2)
O1—H11	0.8479	O24—S2	1.469 (2)
O1—H12	0.8471		
			
C3—C1—N1	116.5 (3)	C5—N1—H1D	107.8
C3—C1—H1A	108.2	C1—N1—H1D	107.8
N1—C1—H1A	108.2	H1C—N1—H1D	107.1
C3—C1—H1B	108.2	C2—N2—C4	115.4 (3)
N1—C1—H1B	108.2	C2—N2—H2C	108.4
H1A—C1—H1B	107.3	C4—N2—H2C	108.4
N2—C2—C3	114.1 (3)	C2—N2—H2D	108.4
N2—C2—H2A	108.7	C4—N2—H2D	108.4
C3—C2—H2A	108.7	H2C—N2—H2D	107.5
N2—C2—H2B	108.7	Co1—O1—H11	111.8
C3—C2—H2B	108.7	Co1—O1—H12	121.0
H2A—C2—H2B	107.6	H11—O1—H12	110.1
C1—C3—C2	115.6 (4)	Co1—O2—H21	121.5
C1—C3—H3A	108.4	Co1—O2—H22	105.3
C2—C3—H3A	108.4	H21—O2—H22	110.8
C1—C3—H3B	108.4	Co1—O3—H31	112.0
C2—C3—H3B	108.4	Co1—O3—H32	115.5
H3A—C3—H3B	107.5	H31—O3—H32	107.6
C5—C4—N2	117.4 (4)	Co1—O4—H41	123.8
C5—C4—H4A	108.0	Co1—O4—H42	124.7
N2—C4—H4A	108.0	H41—O4—H42	108.5
C5—C4—H4B	108.0	Co1—O5—H51	126.2
N2—C4—H4B	108.0	Co1—O5—H52	114.6
H4A—C4—H4B	107.2	H51—O5—H52	104.7
C4—C5—N1	117.5 (4)	Co1—O6—H61	112.4
C4—C5—H5A	107.9	Co1—O6—H62	125.1
N1—C5—H5A	107.9	H61—O6—H62	107.0
C4—C5—H5B	107.9	Co2—O7—H71	115.0
N1—C5—H5B	107.9	Co2—O7—H72	114.0
H5A—C5—H5B	107.2	H71—O7—H72	113.5
O6—Co1—O2	90.18 (10)	Co2—O8—H81	126.0
O6—Co1—O1	93.94 (10)	Co2—O8—H82	122.8
O2—Co1—O1	175.87 (9)	H81—O8—H82	109.6
O6—Co1—O3	177.76 (10)	Co2—O9—H91	113.5
O2—Co1—O3	91.02 (9)	Co2—O9—H92	117.1
O1—Co1—O3	84.85 (9)	H91—O9—H92	110.1
O6—Co1—O5	88.96 (11)	Co2—O10—H101	120.0
O2—Co1—O5	91.94 (10)	Co2—O10—H102	109.8
O1—Co1—O5	88.27 (10)	H101—O10—H102	111.3
O3—Co1—O5	92.89 (10)	Co2—O11—H111	116.4
O6—Co1—O4	91.14 (10)	Co2—O11—H112	124.0
O2—Co1—O4	85.66 (10)	H111—O11—H112	109.4
O1—Co1—O4	94.12 (10)	Co2—O12—H121	108.8
O3—Co1—O4	87.06 (10)	Co2—O12—H122	119.2
O5—Co1—O4	177.59 (10)	H121—O12—H122	108.3
O11—Co2—O9	92.90 (10)	O14—S1—O13	111.44 (15)
O11—Co2—O8	178.79 (10)	O14—S1—O15	110.01 (17)
O9—Co2—O8	88.09 (10)	O13—S1—O15	109.02 (16)
O11—Co2—O10	90.99 (10)	O14—S1—O16	110.02 (14)
O9—Co2—O10	86.67 (9)	O13—S1—O16	109.24 (17)
O8—Co2—O10	89.75 (10)	O15—S1—O16	106.99 (17)
O11—Co2—O12	84.92 (9)	O23—S2—O24	110.93 (15)
O9—Co2—O12	177.80 (9)	O23—S2—O22	110.19 (15)
O8—Co2—O12	94.08 (10)	O24—S2—O22	109.09 (16)
O10—Co2—O12	93.70 (9)	O23—S2—O21	109.37 (15)
O11—Co2—O7	90.08 (10)	O24—S2—O21	108.66 (14)
O9—Co2—O7	93.75 (9)	O22—S2—O21	108.55 (14)
O8—Co2—O7	89.17 (10)	O19—S3—O18	109.6 (2)
O10—Co2—O7	178.82 (9)	O19—S3—O20	109.45 (16)
O12—Co2—O7	85.91 (9)	O18—S3—O20	108.66 (15)
C5—N1—C1	118.1 (3)	O19—S3—O17	109.02 (15)
C5—N1—H1C	107.8	O18—S3—O17	109.01 (17)
C1—N1—H1C	107.8	O20—S3—O17	111.07 (15)

### Hydrogen-bond geometry (Å, °)

**Table:**

*D*—H···*A*	*D*—H	H···*A*	*D*···*A*	*D*—H···*A*
N1—H1C···O24	0.90	1.87	2.758 (4)	171.
N1—H1D···O18^i^	0.90	1.84	2.723 (4)	167.
N2—H2C···O15	0.90	2.00	2.820 (4)	151.
N2—H2D···O21^ii^	0.90	1.99	2.850 (4)	161.
O1—H11···O23^iii^	0.85	1.91	2.738 (3)	165.
O1—H12···O20	0.85	1.93	2.774 (3)	178.
O2—H21···O19^iv^	0.84	1.84	2.682 (4)	176.
O2—H22···O15	0.85	2.00	2.843 (4)	169.
O3—H31···O24^v^	0.85	1.89	2.722 (3)	167.
O3—H32···O16	0.85	2.16	2.982 (4)	163.
O3—H32···O15	0.85	2.53	3.152 (4)	130.
O4—H41···O20^vi^	0.85	1.99	2.840 (3)	175.
O4—H42···O17	0.85	1.90	2.733 (3)	166.
O5—H51···O17^iv^	0.86	2.00	2.855 (3)	175.
O5—H52···O23^v^	0.86	1.88	2.726 (3)	169.
O6—H61···O19^vi^	0.85	1.82	2.665 (4)	178.
O6—H62···O22^iii^	0.85	1.92	2.749 (3)	164.
O7—H71···O15	0.84	2.14	2.908 (4)	152.
O7—H72···O17	0.84	2.00	2.829 (3)	169.
O8—H81···O16	0.85	1.88	2.722 (3)	174.
O8—H82···O13^vii^	0.85	1.88	2.725 (3)	171.
O9—H91···O18	0.85	1.84	2.681 (3)	173.
O9—H92···O14^viii^	0.85	1.91	2.742 (4)	165.
O10—H101···O22^vii^	0.85	1.94	2.788 (3)	174.
O10—H102···O14^vii^	0.84	1.91	2.742 (3)	170.
O11—H111···O21^ii^	0.85	1.92	2.759 (3)	168.
O11—H112···O16^viii^	0.85	1.88	2.729 (3)	174.
O12—H121···O21^vii^	0.85	1.96	2.806 (3)	176.
O12—H122···O13	0.85	1.91	2.728 (3)	162.

Symmetry codes: (i) *x*, *y*+1, *z*; (ii) *x*, −*y*+3/2, *z*+1/2; (iii) −*x*+1, −*y*+1, −*z*; (iv) *x*, −*y*+1/2, *z*−1/2; (v) *x*, *y*−1, *z*; (vi) −*x*+1, *y*+1/2, −*z*+1/2; (vii) −*x*+2, *y*−1/2, −*z*+1/2; (viii) *x*, −*y*+1/2, *z*+1/2.
